# PathOS: a decision support system for reporting high throughput sequencing of cancers in clinical diagnostic laboratories

**DOI:** 10.1186/s13073-017-0427-z

**Published:** 2017-04-24

**Authors:** Kenneth D. Doig, Andrew Fellowes, Anthony H. Bell, Andrei Seleznev, David Ma, Jason Ellul, Jason Li, Maria A. Doyle, Ella R. Thompson, Amit Kumar, Luis Lara, Ravikiran Vedururu, Gareth Reid, Thomas Conway, Anthony T. Papenfuss, Stephen B. Fox

**Affiliations:** 1 Research Division, Peter MacCallum Cancer Centre, East Melbourne, VIC Australia; 2 Department of Pathology, Peter MacCallum Cancer Centre, East Melbourne, VIC Australia; 3 Sir Peter MacCallum Department of Oncology, University of Melbourne, Melbourne, VIC Australia; 4 Department of Pathology, University of Melbourne, Melbourne, VIC Australia; 5 Department of Medical Biology, University of Melbourne, Melbourne, VIC Australia; 6 Bioinformatics Division, The Walter and Eliza Hall Institute of Medical Research, Parkville, VIC Australia; 7 Children’s Cancer Institute, University of New South Wales, Sydney, NSW Australia; 8 Peter MacCallum Cancer Centre, 305 Grattan Street, Parkville, VIC 3000 Australia

## Abstract

**Background:**

The increasing affordability of DNA sequencing has allowed it to be widely deployed in pathology laboratories. However, this has exposed many issues with the analysis and reporting of variants for clinical diagnostic use. Implementing a high-throughput sequencing (NGS) clinical reporting system requires a diverse combination of capabilities, statistical methods to identify variants, global variant databases, a validated bioinformatics pipeline, an auditable laboratory workflow, reproducible clinical assays and quality control monitoring throughout. These capabilities must be packaged in software that integrates the disparate components into a useable system.

**Results:**

To meet these needs, we developed a web-based application, PathOS, which takes variant data from a patient sample through to a clinical report. PathOS has been used operationally in the Peter MacCallum Cancer Centre for two years for the analysis, curation and reporting of genetic tests for cancer patients, as well as the curation of large-scale research studies. PathOS has also been deployed in cloud environments allowing multiple institutions to use separate, secure and customisable instances of the system. Increasingly, the bottleneck of variant curation is limiting the adoption of clinical sequencing for molecular diagnostics. PathOS is focused on providing clinical variant curators and pathology laboratories with a decision support system needed for personalised medicine. While the genesis of PathOS has been within cancer molecular diagnostics, the system is applicable to NGS clinical reporting generally.

**Conclusions:**

The widespread availability of genomic sequencers has highlighted the limited availability of software to support clinical decision-making in molecular pathology. PathOS is a system that has been developed and refined in a hospital laboratory context to meet the needs of clinical diagnostics. The software is available as a set of Docker images and source code at https://github.com/PapenfussLab/PathOS.

**Electronic supplementary material:**

The online version of this article (doi:10.1186/s13073-017-0427-z) contains supplementary material, which is available to authorized users.

## Background

The transition from single gene assays to multiple cancer gene panels has highlighted the need for scalable reporting systems capable of supporting increasing assay volumes.

Clinical diagnostics often involves a complex chain of technology, software and expertise interoperating to achieve a robust, clinically defensible report. Increasingly, computer software and databases are involved in expanding the scope, accuracy and detail of diagnostic assays. Clinical cancer next-generation sequencing (NGS) assays, in particular, are dependent on many software subsystems and databases to deliver their results. The authors have previously highlighted a number of issues surrounding these dependencies [1] and in this paper we present a solution to address the lack of decision support tools in molecular diagnostics.

The Peter MacCallum Cancer Centre (Peter Mac) is Australia’s largest hospital dedicated to cancer. The Department of Pathology at the Peter Mac performs a wide variety of assays for internal patients as well as regional and national pathology laboratories. In 2012, the lab undertook a transition from traditional Sanger-based DNA sequencing to higher volume NGS allowing multiple genes and multiple samples to be routinely sequenced in a single sequencing run. Additionally, a large prospective pan-cancer study [2] required the storage and analysis of thousands of research samples and their variants in addition to operational patient samples. At this time, the lack of software applications capable of storing, analysing and reporting on NGS variants led to the development of the in-house system described in this paper.

The intrinsic nature of cancer highlights many challenges for sequencing. Germline samples contain homozygous and heterozygous variants present at allele frequencies of 100% and 50%, respectively. These values are well above the background level of low-frequency sequence variants associated with sequencing errors typical of NGS. In contrast, tumour specimens can contain an unknown mix of tumour and non-tumour cells, resulting in reduced variant allele frequency. Additionally, tumour heterogeneity can further dilute the mutational signal of variants.

The need to reliably identify low-frequency somatic variants has led Peter Mac to employ targeted deep sequencing of samples via custom and off the shelf amplicon panels or targeted capture panel technology. In contrast to whole-exome or whole-genome sequencing, this allows high sensitivity through very deep sequencing (>1000× coverage) across cancer implicated genes and mutational hot spots.

A consequence of building software systems for clinical use is the mandatory requirements of reliability and reproducibility imposed by diagnostic laboratory accreditation bodies such as Clinical Laboratory Improvement Amendments (CLIA), National Association of Testing Authorities (NATA) [3] and the International Organisation for Standardisation (ISO 15189). In addition to regulatory obligations, medical systems storing patient level genetic data should operate as an operationally critical system and encompass functionality such as password protection, role-based access, audit trails, high availability and version controlled release cycles. Many of these features are not found in research software, the common pedigree of genomics software.

The adoption of NGS in a clinical diagnostic setting has highlighted the need for laboratories to automate previously manual processes. This trend will continue as the demand for more complex assays increases and improving technology allows patients to be tested multiple times during their health system encounter with techniques such as liquid biopsies [4]. The last few years have seen many software systems appear which assist in the automation of NGS assay validation, analysis, curation or reporting. Of these, few can perform all these tasks and the majority of these are commercial packages [5–10].

A review of the non-commercial systems highlights the diversity of approaches used by NGS analysis groups. Some systems focus on the web presentation and filtering of VCF files but without the ability to curate and report variants [11, 12]. There are systems that focus on translational research and the analysis or exploration of large datasets (such as TCGA) but not the reporting of patient clinical results [13–18]. For a review of publicly available research platforms, see this paper [19]. Clinical trial reporting has also given rise to systems for the management of large cohort trials but these lack clinical reporting capabilities [20, 21]. The need to curate variants in a gene-centric fashion has produced locus-specific database (LSDB) systems [22, 23] but again without clinical reporting facilities. There are also Mendelian disease-focused systems [24, 25] unsuitable for cancer diagnostics. In the area of open access web resources for cancer variant evidence, the contribution of CIViC [26] is a significant and valuable resource. Future releases of PathOS will enable compatible data exchanges with CIViC to leverage the community knowledgebase it represents. More complete systems that appear suitable for clinical reporting of NGS assays are from Emory Genetics [27] and from the University of Pittsburgh [28] but neither of these appear to be publicly available.

Interestingly, a survey of seven of the largest genetic laboratories in the US [29] identified that all had developed in-house systems for the analysis, curation and reporting of NGS assays and were not using commercial tools apart from Brigham and Women’s Hospital–Harvard Medical School who use GeneInsight [10]. Significantly, none of these institutions are making their systems available for public use. This leaves less resourced diagnostic laboratories around the world with difficult choices. Either they attempt to develop complex decision support systems in-house and keep them current in a rapidly changing environment or they purchase a commercial license and rely on the vendor’s product meeting the needs of their assays and laboratory integration requirements. PathOS addresses this lack of clinical-quality NGS decision support systems with a web application that can ingest results from a bioinformatics pipeline generating compliant VCF [30] files and manage the pathology laboratories workflows through to a professional clinical genetics report. The current focus is on amplicon and capture panel assays rather than whole-genome sequencing (WGS) as these assays currently have the most clinical utility for high volume cancer diagnostics, although the system can operate with any valid VCF pipeline data. The genesis of PathOS has been for the clinical reporting of cancer samples; however, the filtering, curation and reporting of any NGS data can be performed by the system. The scaling of PathOS to WGS scale analysis is only limited by underlying database performance. The current data storage platform is MariaDB [31] (a MySQL compatible DB, as used by Google®).

This paper describes PathOS’ features and workflows incorporating variant filtering, curation and reporting and their integration into a complete system.

## Implementation

The processing of patient samples through to a clinical report involves wet lab, bioinformatic and analysis steps [1]. PathOS addresses the analysis and reporting steps of the process but should be viewed in the context of an entire diagnostic ecosystem.

**Fig. 1:**
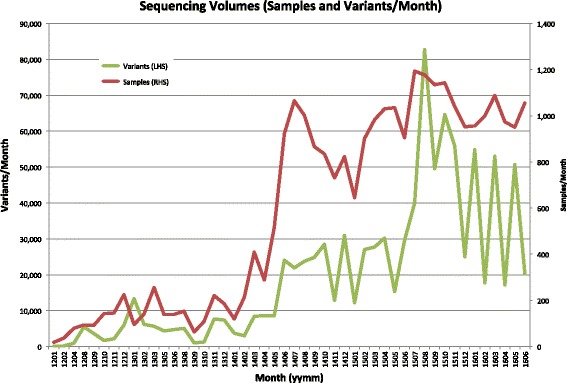
Sample and variant volumes. *Chart* of the increase of sample and unique sequenced variants by month from January 2012. 2016 cancer diagnostic volumes for the Peter MacCallum Molecular Diagnostic Laboratory were 151 sequencing runs of 6023 samples yielding 213,581 unique variants

The following sections describe features of PathOS from a workflow perspective. PathOS currently supports a number of commercial assays and custom panels for a variety of tumour streams. The respective volumes of these assays are shown in Table 1. Since the introduction of PathOS in 2013, the volume of assays processed has grown at approximately 26% per month. In addition to the clinical reporting of the Pathology Department, a research instance of PathOS has been used to manage variants from a number of clinical trials and research projects [2, 32–34] (see Fig. 1). Current variant types supported include single nucleotide variants (SNV) and small insertions and deletions (indels). Copy number variants (CNV) are also displayed from the upstream pipelines and new features are actively being developed, such as support for structural variants (SV) and mutational signatures. A schematic of the end-to-end workflow is shown in Additional file 1: Figure S1 and in an earlier paper (Supplementary Figures) [1].

**Table 1:** Diagnostic assay types

Assay	Origin	Type	Description (genes)	Size of panel (bases)	Sample volumes (up to June 2016)
Germline	Custom in-house	Amplicon	Predictive and diagnostic panel for routine germline assays (4)	28.6 Kb	7822
Somatic	Custom in-house	Amplicon	Multiple tumour stream panel for routine somatic assays (16^a^)	18.4 Kb	4325
Myeloid	Custom in-house	Amplicon	Myeloid panel for routine haem. assays (26^a^)	29.9 Kb	1311
Lymphoid	Custom in-house	Amplicon	Lymphoid panel for routine haem. assays (21^a^)	20.0 Kb	495
Clinical trials	Illumina	Dual strand amplicon	Panels for volume clinical trial (41)	26.4 Kb	1323
Clinical cancer panel	Custom in-house	Hybrid capture	General purpose somatic cancer gene panel for routine clinical use (391^a^)	2.34 Mb	343

^a^Targeted at gene hotspot regions

### Sequencing

**Fig. 2:**
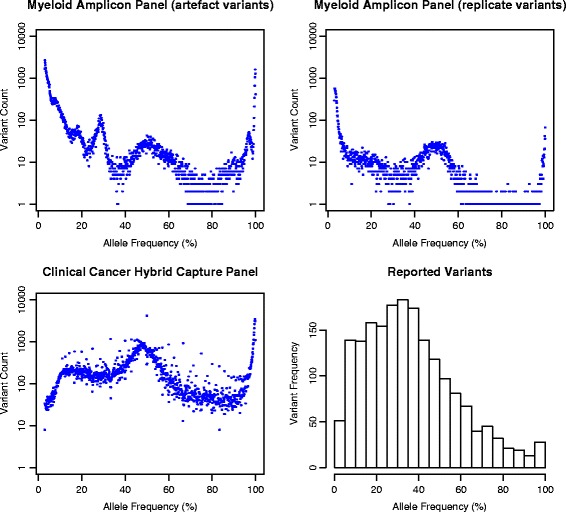
Variant allele frequency (VAF) distributions. The variant data for the first six months of 2016 have been aggregated to show the VAF distributions for amplicon and hybrid capture panels. All *scatter plots* display a bimodal distribution with a peak at 50% allele frequency for heterozygous variants and 100% for homozygous variants. The *top left plot* shows all variants in the custom myeloid amplicon panel prior to filtering (*n* = 66,210). It shows a number of peaks that are due to technical panel artefacts. The *top right plot* shows variants remaining (*n* = 13,649 20.6%) after removing; variants occurring in one sample replicate only, variants occurring in more than 35% of samples in the myeloid panel (panel artefacts) and variants with less than 100 total reads or less than 20 alternative reads. The resulting distribution is far smoother and free from technical artefacts. Note the large peak at low VAF%. The amplicon panel samples have high read coverage (mean 2297×) which captures low frequency variants from both the wet lab PCR processes and sequencer errors. In contrast, the bottom left plot shows variants from the hybrid capture cancer panel and has no low VAF peak (mean coverage 246×). This is due to multiple factors including lower coverage meaning fewer low VAF variants pass the variant caller threshold (3.0%), more stringent pipeline filtering for hybrid capture and different wet lab processing. The histogram shows all manually reported somatic variants over this period and shows a skew towards low VAF% due to tumour purity (samples of mixed tumour and normal cells) and tumour heterogeneity (variants occurring only within clones in a heterogeneous tumour)

The Peter Mac Molecular Pathology Laboratory registers patient samples from within the hospital and from external pathology labs. These are usually formalin-fixed paraffin-embedded (FFPE) solid tumour samples for somatic assays or blood samples for haematopathological or familial cancer assays. Samples are processed to DNA by a Hamilton liquid handling system under the control of in-house and LIMS software. This process extracts and quantitates DNA prior to polymerase chain reaction (PCR) amplification in preparation for sequencing. Somatic samples are sequenced as technical replicates to control for the false-positive rate inherent in amplicon-based NGS. The amplicon panel samples have high read coverage (mean 2297×) which captures low frequency variants from both the wet lab PCR processes and sequencer errors (Fig. 2). PathOS flags variants that appear in only one replicate and these may be filtered from subsequent processing. Typical somatic sequencing runs contain 22 patient samples, NA12878 [35] control samples and non-template controls, making a total of 48 samples per sequencing run.

Sequencing the production targeted somatic assay on an Illumina MiSeq instrument typically yields around 50 million reads at a median coverage depth of 3800×. A number of quality control (QC) metrics are collected from the sequencing and pipeline processes such as total reads, unmapped reads and poor coverage regions. A number of common bioinformatic tools are used which generate QC data and are detailed in Table 2.

**Table 2:** Pipeline dependencies

Tool	Version	Description	Link
Bpipe	0.9.8	Pipeline workflow framework	http://download.bpipe.org/
vt	1.0	Vcf manipulation tool set	http://genome.sph.umich.edu/wiki/Vt
Igvtools	2.3.72	IGV tools, used for indexing VCF files for use by IGV	https://www.broadinstitute.org/igv
Fastqc	0.10.1	Fatsq file quality assessment tool	http://www.bioinformatics.babraham.ac.uk/projects/fastqc
Samtools	0.1.18	BAM and other file manipulation tool	https://sourceforge.net/projects/samtools
VarScan	2.3.3	Variant caller for SNPs and indels	http://sourceforge.net/projects/varscan
Gatk	3.4	Genome analysis toolkit from Broad Institute	https://software.broadinstitute.org/gatk
Primal aligner	1.01	In-house developed amplicon aligner in Perl	
Canary	0.9	In-house developed amplicon aligner and variant caller in Java	Manuscript in preparation
NormaliseVcf	1.2	In-house VCF normalisation tool for annotating VCFs with gene, transcript and HGVS nomenclature	Manuscript in preparation
Picard	1.141	Tools for manipulating high-throughput sequencing (HTS) data	http://sourceforge.net/projects/picard
Ensembl DB	78 - 85	Annotation and consequences database	http://www.ensembl.org
Bcl2fastq	2.17.1	Illumina BCL to fastq file convertor	https://support.illumina.com/sequencing/sequencing_software/bcl2fastq-conversion-software.html

The upstream amplicon pipeline has a number of external tool dependencies which are shown in this table

**Fig. 3:**
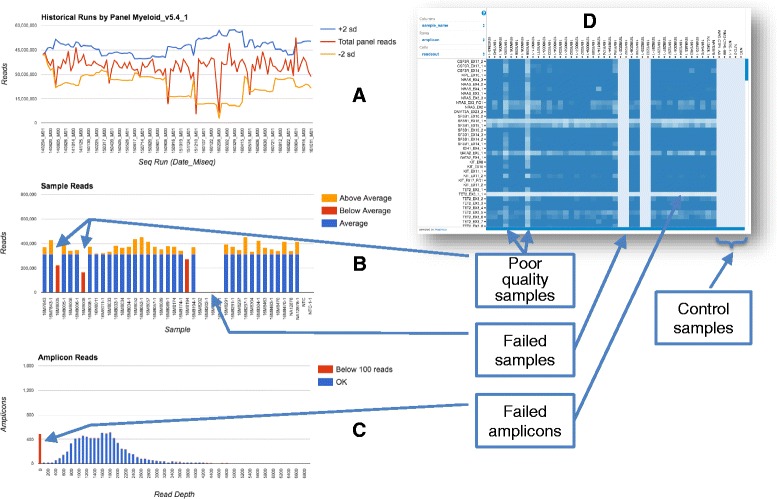
Quality control of runs and samples. Screen shots of graphical quality control metrics. Quality control is monitored at the sample, sequencing run and amplicon level. **a** A sequencing run’s read yield is compared to all previous runs of the same assay and should reside between ± 2 standard deviations for the last ten runs. Failed runs can be seen here dropping below the lower bound. **b** All samples within a run can be compared and samples with below average reads are highlighted in *red*. **c** The per amplicon reads over all samples in the run are binned and graphed to highlight their distribution and highlight any amplicons with less than 100 reads. Non-template controls are included in each run and are flagged if they contain any reads. Both a sequencing run and samples within the run must be QC passed or failed by the user prior to curation reports being produced. **d** The configurable *heatmap* of number of reads by amplicon and sample. *Lighter horizontal bands* indicate poorly performing amplicons while *lighter vertical bars* show poorly sequenced samples, typically due to insufficient or fragmented sample DNA

The total reads per run metrics are used to compare the current run to historical runs of the same assay. The total reads generated should fall within ± 2 standard deviations of the previous ten runs (derived from the Westgard rules for clinical validity). Graphs are displayed on the run QC screen are indicators of run, sample and assay quality (Fig. 3). The software does not pass or fail runs or samples, but the user must determine this from multiple displayed metrics in conjunction with the standard operating procedures (SOP) for laboratory sequencing.

### Pipelines

Although the clinical pipelines used for production sequencing is not part of the PathOS web application, it is versioned in the Bitbucket [36] source code control system and shares the same test and release cycles (see “Software Deployment” section). This alignment ensures that the data loaded into the system matches the expected fields and semantics. The bioinformatic pipelines are matched to the assay types and have been validated as part of the NATA [3] accreditation to the ISO 15189 (Medical Laboratories) standard. Each new PathOS release undergoes a regression test cycle to ensure that any software changes will not impact the data processing of the system.

The pipeline operation in PathOS has been implemented in the Bpipe framework [37]. This allows pipelines to be constructed and tested in a modular fashion and allows rapid adoption of new technology and bioinformatic tools into clinical assays. Within the pipeline stages, all third-party tool dependencies and parameters are explicitly defined to prevent inadvertent tool version changes outside of PathOS release cycles. This ensures overall integrity of assay performance in which PathOS and the pipeline participate. The Amplicon pipeline tools are described in Table 2. Additional tools are used in hybrid capture pipeline and are currently being detailed (manuscript in preparation).

### Variant shifting and nomenclature

All SNVs and indel variants are stored in PathOS and identified using genome build hg19 (GRCh37) and HGVS [38] nomenclature. Although hg19 is not the latest genome build, clinical nomenclature and much recent medical literature uses this build. Future software versions will need to accommodate both hg19 and GRCh38/hg38 as newer literature adopts the later build in its nomenclature. To remove multiple representations of the same variant, the variants undergo a normalisation process. Multi-allele variants are split into their constituent parts and then all variants are trimmed to their simplest representation and left shifted along the chromosome if possible [39]. PathOS maintains a table of all transcripts for genome builds together with their exon positions. Administrators may assign a reportable transcript for each gene, as determined by the scientist responsible for the assay, and is usually the transcript most frequently cited in clinical literature or reported in variant databases. This may not always coincide with the longest transcript denoted as the canonical transcript by Ensembl. Variants occurring within reportable transcripts are further normalised by shifting towards the 3’ end of the gene if possible, in line with HGVS standards. This process takes advantage of the Mutalyzer SOAP API [40] and also assigns an HGVSc and HGVSp annotation and changes insertions (ins) to duplications (dup) if required. Any 3’ shifted variants also have their HGVSg positions adjusted. A similar normalisation is applied to variants imported from external data sources such as global variant databases. Normalisation is the key to ensuring that sequenced variants may be unambiguously matched to variants in global knowledge bases and be appropriately annotated.

### Annotation

The variants identified in a sequencing run are annotated as a single group for efficiency. A typical run of 24 samples against a 30 kb amplicon panel (on an Illumina MiSeq) will usually yield 50 million reads with a mean of 101 variants per sample while a 568 cancer gene capture panel (on an Illumina NextSeq) covering 3 Mb yields 600 million reads and a mean of 5750 variants per sample.

Samples and their variants are batched when a sequencing run completes. Because samples within a run often have many common variants (recurrent assay artefacts, common polymorphisms), it is efficient to annotate all run variants as a batch and only annotate distinct variants within the batch. Additional efficiencies are gained by caching annotations so that they do not need to be reannotated when seen in subsequent runs. Over a recent two-week period (13 sequencing runs), the median percentage of distinct variants was 30.4%. Of these, a median percentage of 13.5% variants were novel to previous annotations cached within the PathOS database. The caching of annotation data and aggregated variant annotation over this period gave a 24-fold reduction in annotation time per run (the median percentage of variants needing annotation for all runs was 30.4% × 13.5% = 4.1%).

Annotated variants are cached and keyed by data source which currently includes VEP [41], Annovar [42, 43], Mutalyzer [38], Clinvitae [44] and IARC [45]. These data sources in turn aggregate a number of other sources such as Clinvar [46], kConFab [47] and COSMIC [48–50]. In addition to speeding up variant processing, caching facilitates independently refreshing each data source and is currently performed as part of a PathOS regression testing release cycle. Each data source contains varying numbers of attributes for each variant. These attributes are not normalised by the system but maintained together with metadata, which categorises and describes each attribute. The attribute metadata also contains a customisable list of tags to allow users to search for specific types of variant attributes or customise their screen information during data review.

### Filtering

There are two mechanisms for filtering annotated variants produced by the pipelines. The first is automatically applied by PathOS at data load time based on the assay being performed while the second is user selectable through the web GUI. In the second case, the user is permitted to report on any variant regardless of its filtered state. Appropriate workflow processes are enforced to match laboratory practices (see “Curation” section).

**Fig. 4:**
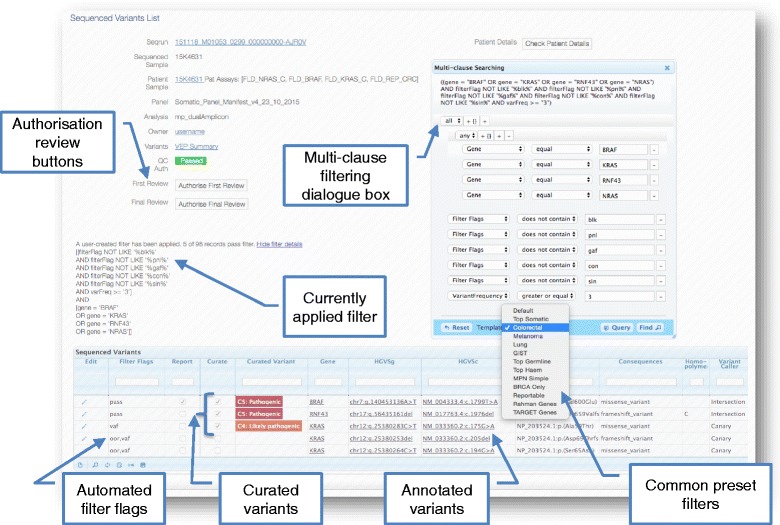
User filtering of variants. *Screenshot* showing multi-clause filtering dialogue box. Users can construct complex multi-clause filters from over 70 variant attributes or choose from common preset filters. PathOS automatically applies one or more flags (when uploading samples) to each variant based on its annotations. These flags are available for user filtering as shown in the filter being applied in the screen shot. The flags are listed with typical filtering criteria in parenthesis: **pass**: Passed all filters. **vaf**: Low variant allele frequency (<8% Somatic, < 15% Germline). **vrd**: Low total read depth (<100 reads). **vad**: Low variant read depth (<20 reads). **blk**: Assay specific variant black list (user defined). **oor**: Out of assay specific region of interest (user defined). **con**: Inferred benign consequences (system defined). **gmaf**: High global minor allele frequency (>1%). **pnl**: Frequently occurring variant in assay (>35%). **sin**: Singleton variant in replicate samples (not in both samples)

Multiple in-built filtering flags are applied to each variant when it is loaded into PathOS at the completion of a sequencing run. A filtering configuration file contains threshold parameters for each assay including minimum variant depth, minimum read depth, minimum variant frequency, maximum variant frequency for samples within an assay and a blacklist of variants for the assay. These automatic filtering flags are described in Fig. 4.

In addition to the filtering of variants based on these automatically applied flags, the user may also apply complex multi-clause filters based on any of 93 annotations associated with each variant (Fig. 4, Additional file 2: Table S1). Specific gene sets can be selected by the user with clauses such as:

*[Gene ‘Is In’ BRAF,KRAS,RNF43,NRAS]* where the gene list may be hundreds of genes long.

A number of preset filter templates allow filters to be applied for particular clinical scenarios. For example, choosing the “Colorectal” filter will display all variants in the genes BRAF, KRAS and NRAS that are not blacklisted, occur in both replicates, have inferred protein coding consequences, have < 1% allele frequency in population databases and do not occur frequently in other assay samples. Users can inspect these filtered variants first as these are most likely to include reportable variants. Other preset filters and their genes include: Melanoma (BRAF, NRAS, RAC1, KIT); Lung (BRAF, EGFR, KRAS, MET); Gastro Intestinal Stromal Tumour (KIT, PDGFRA); BRCA Only (BRCA1, BRCA2); and MNP Simple (JAK2, MPL, CALR, KIT, SF3B1, CSF3R, ASXL1). There are also filters for large gene sets such “Rahman Genes” [51] and TARGET Genes [52].

PathOS is used for routinely reporting germline and somatic samples. The automatically applied filters and the preset filter templates differ for these two sample types: germline panels are configured with higher minimum VAF% threshold (15%) and the panel blacklists reflect known germline polymorphisms as well as poor sequencing regions such homopolymer regions. A zygosity column is displayed for germline assays only on the tabular variant page flagging variants as heterozygous, homozygous or other.

The tabular columns of sample variants can be customised and saved by users to suit their needs. Columns can be reordered by dragging the headers left or right or hidden if not needed. A user may save their customised layout in their personal settings, however, once a sample has passed first review, the variant filters and column layouts are fixed to prevent other scientists from missing key attributes of the data. Any of the annotated attributes are available for display for each variant. All tabular data may be exported from the system as either a CSV or MS Excel file.

### Curation

The data analysis workflow within PathOS is dictated by the laboratory’s standard operating procedures (SOP) and reflects common practice within diagnostic laboratories. PathOS supports role-based access controls (RBAC) assigned by username. RBAC applies for both page level access and also at a more granular level within pages by controlling which actions can be performed by that role. The currently defined roles and descriptions are shown in Table 3.

**Table 3:** User roles

Role	Description
ROLE_ADMIN	• Full system access • Create and remove users • Assign user roles
ROLE_DEV	• Same rights as ROLE_ADMIN except, • Patient names and DOBs are suppressed on screen and in reporting • Additional diagnostics are available within all environments (including Production)
ROLE_CURATOR	• The curators can create variant evidence • Assign the pathogenicity for curated variants. • If all checks pass they can lock a sample into the final review state • Produce final reports
ROLE_LAB	• The user can update the Seqrun QC • Update Sample QC • Submit variants for curation • Submit a sample for first review • Produce draft reports
ROLE_EXPERT	The user has the same access as a curator but can subscribe to certain curation categories of interest such as genes, variants or patients
ROLE_VIEWER	The user only has access to the splash page and the reference tables

PathOS supports multiple roles for access control and workflow

**Fig. 5:**
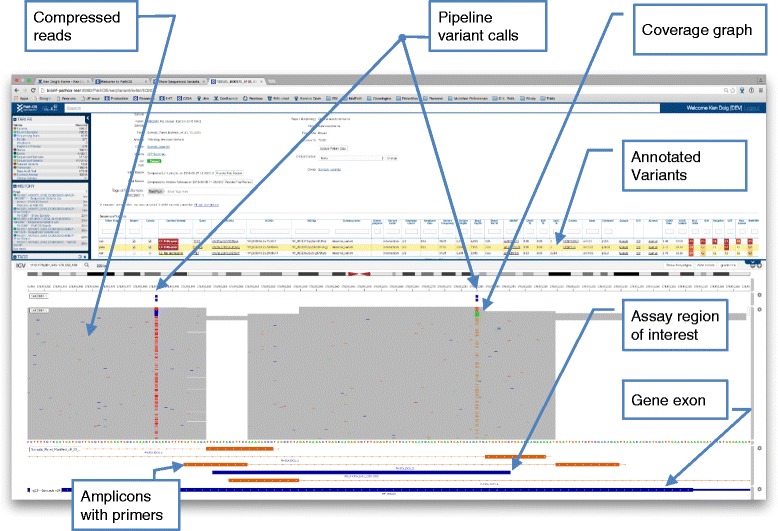
Validating variants with the embedded genome browser. PathOS links directly to the highlighted variant locus in the browser and preloads the correct tracks for reads, variants and amplicon tracks

The standard laboratory workflow within PathOS is for a run to be assessed for quality control (QC), and if accepted, QC of samples is undertaken. Individual samples must then be assessed using alignment metrics such as percent mapped reads, unmapped reads, low read amplicons and read quality as determined by FASTQC [53]. Both run and sample QC must be passed by a laboratory user. The analysis of variants from an individual sample takes place on a page displaying patient details, assay requested and review status. All variants found by sequencing and associated with a preferred transcript can be displayed, irrespective of which in-built filter flags are set. The filter flags assist the scientist to identify variants of likely relevance to the patient’s cancer. At any time, the scientist can inspect the raw reads in the region of a variant by viewing them with the embedded genome browser [54] (see Fig. 5). Alternatively, users can click on a link to an external IGV [55] instance which loads a PathOS-generated IGV session file for each sample. For both actions, the current variant is displayed in context using the pipeline generated BAM, BED and VCF files and shows relevant tracks such as amplicon locations for the regions targeted by the assay. The pipeline data repository used by the in-built browser or IGV is served by an Apache web instance. The architecture of the system allows for the database, the pipeline data repository and the PathOS website to be located on different servers or in different institutions as required. Once inspected, variants can then be selected for curation and optionally reporting by the lab scientist. Curation refers to the expert interpretation of sequence variants in the clinical context in which they present. When complete, the “Authorise First Review” button is clicked to change the sample workflow status. A second review stage can then be authorised which, optionally, can generate a work ticket in the laboratory issue tracking system to notify the curation team. The Peter Mac laboratory uses Atlassian JIRA [56] for tracking variant curation operations, but the interface is customisable for other issue tracking systems. Integration of PathOS workflows with JIRA provides a framework for managing and documenting curation activities.

**Fig. 6:**
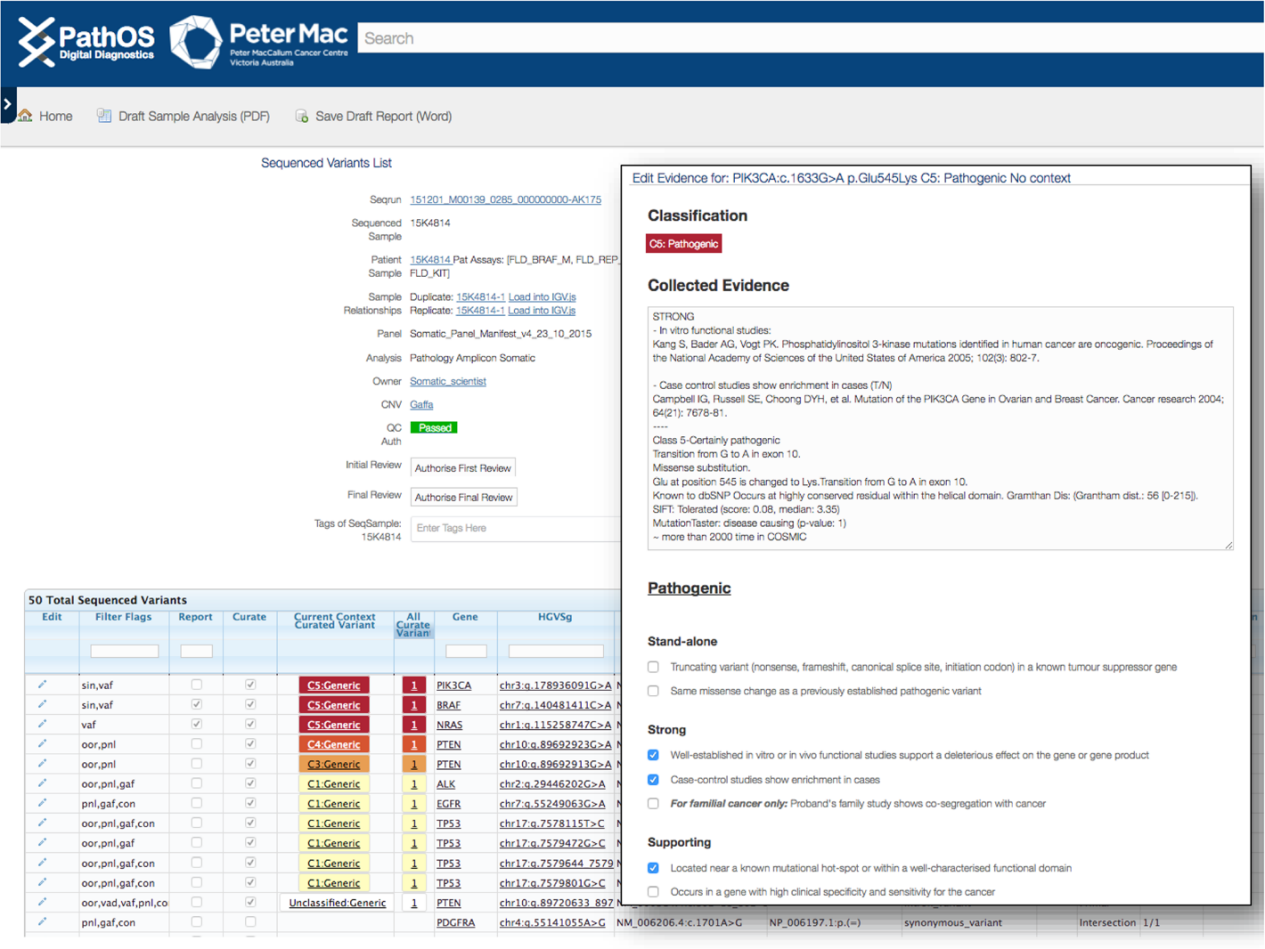
PathOS *screenshots* showing the curation workflow. The curator navigates to the screen on the left displaying all variants (filtered and unfiltered) for a sample. Using an existing search template or a user configurable search dialogue, high priority variants are selected for curation. Previously curated and known variants are shown at the top of the list together with their classification. New variants can be added to the curation database by selecting the “Curate” checkbox. The curator then selects from a set of evidence checkboxes (*right screen*) characterising the mutation. Details are displayed when the mouse hovers over the checkbox to guide the curator’s selection. When the evidence page is saved, the five-level classification is automatically set as adapted from the ACMG guidelines for classification of germline variants

The PathOS users with a curation role may create persistent curation variant records within the database. These records are independent of sequenced variants and are unique for each variant (recorded using HGVS [38] nomenclature) and optionally differentiated by patient disease context. For example, the BRAF V600E has a different clinical consequence when detected in colorectal cancer than in melanoma and should be curated distinctly for each context. Curated variants records need only be created once within PathOS. Once created, all subsequent samples containing the variant and patient condition will be automatically matched with the persistent curation record and its corresponding evidence (Fig. 6).

The curation of germline variants differs from somatic variants in the genes assayed, reference databases used (BIC [57], IARC TP53 [58]) and the artifact criteria used in filtering. In addition, germline curation must account for inheritance mode and familial genetics such as co-segregation with disease. PathOS provides germline-specific criteria on the curation evidence page (Fig. 6).

Curating novel variants can be routine for common types (e.g. a frame-shift mutation in a known tumour suppressor) to complex (e.g. a missense mutation in rare cancer gene with no associated literature). Acquiring the necessary curation evidence from websites, literature and clinical studies takes 0.5–5 h making high-quality curation the limiting factor within diagnostic laboratories. These figures are in line with previous studies [59] highlighting the difference in effort between well understood genes and their variants and less-studied genes.

**Fig. 7:**
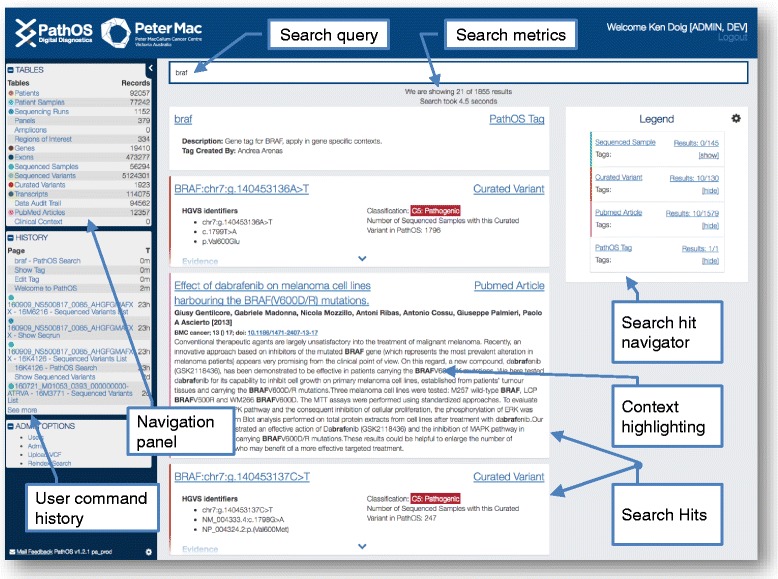
Search results page. Key fields within PathOS objects are designated to be globally searchable by the integrated Apache Lucene search engine. This allows users to easily retrieve the main PathOS data objects: patients, samples, sequenced variants, curated variants, PubMed articles as well as user and system-defined tags. Matching text is highlighted showing the context of the search string within the hits. This *screenshot* shows hits found within PathOS for the string “braf”
- Richly annotating all variants with inferred consequences, currently including nine in silico prediction algorithms and ten global variant knowledge bases which in turn aggregate additional databases (Additional file 2: Table S1),
- Providing links to existing global data sources including prebuilt Google® searches,
- Calculating pathogenicity classifications based on ACMG guidelines for germline variants,
- A searchable PubMed database of over 12,000 cancer specific literature linkable to article PDFs. The initial load of PubMed data was from articles cited within the COSMIC database of somatic variants. Users may optionally attach an article’s PDF to the database for ease of access but the system does not retrieve PDFs from external sources due to publication restrictions.

PathOS expedites this effort through a number of strategies:

- Matching sequenced variants with the existing PathOS curated knowledge base,
- A powerful search facility returning context sensitive results for data within system. Users may perform a free text search on the main PathOS data objects: patients, samples, sequenced variants, curated variants, PubMed articles as well as user and system-defined tags. Matching text is highlighted showing the context of the hits (Fig. 7).

Due to the effort and expertise required to curate variants effectively, supporting the curation process is a key focus of future PathOS development efforts.

Once variants are curated, the curator can then pass the sample and its documented variants through to the “Final Review” stage. At this point, final reports may be generated with the findings.

### Reporting

**Fig. 8:**
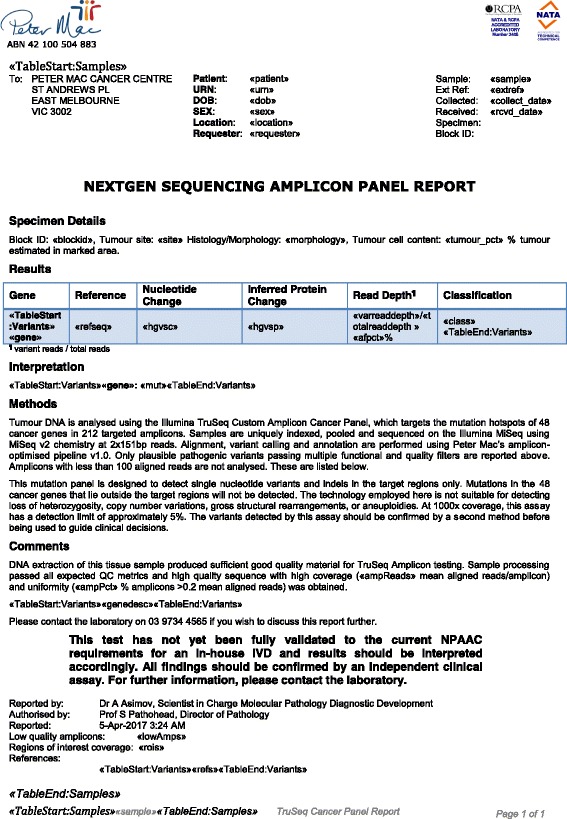
Example MS Word template clinical report. An example of the MS Word mail merge style template that can be used for the format of PathOS clinical reports. Any Word template containing the fields matching PathOS database content may be used for a report template. PathOS with populate the report from patient, sequencing and curation data in PDF or MS Word format when users click on the generate draft report button

There is a wide range of diagnostic reporting preferences for diagnostic labs and even within labs. The reporting requirements for research clinical trials are very different again than for a specific clinical assay. To encompass this range, PathOS passes a defined set of values from the database into the reporting module, which is responsible for the rendering of that information. Each assay is a member of an Assay Group, which has an associated reporting template in MS Word. The template can be formatted in any manner and can include any of the merge fields representing data passed from PathOS (Fig. 8 and Additional file 3). The reporting engine can render the template, incorporating the data, as a PDF file, a Word document or HTML. Current practice within the laboratory is to archive generated reports into the Hospital Pathology LIMS system. PathOS also archives previous reports allowing users to view them for comparison with generated reports. The number of variants in a patient’s report depends on many factors such as the number of genes in the assay, mutational burden and type of cancer. A greater number of less studied genes in a targeted gene assay will increase the curation effort and turnaround time to achieve a clinically acceptable result.

### Platform

PathOS has taken advantage of many open-source and public Java libraries to implement an enterprise-grade application suitable for hospital use and secure storage of patient medical data. It interfaces to laboratory LIMS systems for input of patient demographic details and sample and assay registration data. An HL7 interface is currently being developed to interface with hospital records systems.

The web application is implemented in Java, Javascript, Groovy [60] and Grails [61] deployable on any server supporting java servlet containers such as Tomcat. This allows for deployment in a wide range of environments. Access to the system is controlled by the Spring Security Library which optionally uses an organisations LDAP server for authentication or the internal database for authorisation and role assignment. Web traffic is monitored by Google® Analytics to monitor user activity for workflow and user interface refinement.

The backend database is implemented with MariaDB, a MySQL-compatible relational database, which stores the variant annotation cache and persistent java objects via Spring and Hibernate. The code base is managed in Atlassian Bitbucket on an instance outside the organisation firewall allowing distributed developer access via Git. In addition, a GitHub repository is maintained for public access (https://github.com/PapenfussLab/PathOS). Build management uses Gradle to build system modules and create shared artefacts such as JARs, WARs and TAR files. Atlassian Bamboo is used to perform builds of system modules triggered by developer commits to the code repository. The PathOS search engine is implemented in Apache Lucene. This is a powerful search framework allowing customised search capabilities over any text field in the PathOS domain model.

Project management uses JIRA for issue tracking while project and laboratory documentation is held in Confluence. All Atlassian products used within Peter Mac have been made available through a Community License for not-for-profit organisations from Atlassian.

### Continuous integration and deployment

PathOS developers can operate both within the hospital intranet and/or externally. Code commits to the main development branch trigger an automatic build in Bamboo which then runs 293 unit tests (April 2016). If successful, deployment artifacts are created and automatically deployed to the development environment and the WAR file is deployed to the test Tomcat server. Automatic test execution of the main development branch gives early notice of any code errors or build conflicts. The availability of an up-to-date development instance of the application allows all stakeholders to assess progress and provide early feedback on functionality. Deployment of signed off releases after user acceptance testing (UAT) to the production environment uses the same build and deploy processes as continuous integration to ensure consistent build states.

### Deployment environments

Multiple independent instances of PathOS with their own databases have been deployed to meet the requirements of a number of external stakeholders. The same code base is used for all environments and deployment behaviour, such as file locations and server names, is controlled by a properties file. The main production server is used by the hospital Molecular Pathology Laboratory (35 users) for clinical operations. The hospital also supports over 400 researchers and a research instance of PathOS is provided for research samples. A development server is used for CI and also serves as a UAT platform when releasing new versions.

PathOS has been deployed on Amazon cloud nodes for organisations without the resources to support in-house IT infrastructure, as well as the demonstration PathOS instance. A cloud instance of PathOS has been made available to the Zero Childhood Cancer Program headed by Children’s Cancer Institute at UNSW, Sydney for a multi-Institutional collaboration studying paediatric cancers [62].

## Results and discussion

**Fig. 9:**
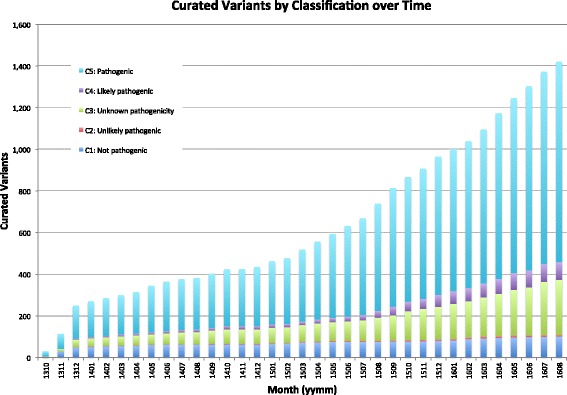
Curated variants by classification over time. This histogram shows counts of the number of curated variants added to PathOS by manual curation by month over the life of the system. Variants are broken down by pathogenicity classification showing a predominance of pathogenic variants due to the focus of clinical sequencing to find disease-causing mutations

PathOS has been used operationally in the Peter MacCallum Cancer Centre since July 2013 for the analysis, curation and reporting of genetic tests for cancer patients as well as the curation of large-scale research studies. As at May 2016, a total of 978 sequencing runs have been processed, comprising 37,651 patients and yielding 3,856,446 variants, of which 297,652 are unique. The curated biological variants within the system number 1068 and are a mixture of germline and somatic. They have been manually curated and classified as “Pathogenic” (797), “Likely pathogenic” (63), “Unknown pathogenicity” (176), “Unlikely pathogenic” (8) and “Not pathogenic” (94) (Fig. 9). Variant curation involves reviewing the automatic variant annotations, assessing the inferred mutational consequences and searches of clinical literature. Each curated variant contains evidence to support the classification and links to literature (if available) and a description of the variant, which is automatically embedded in system-generated reports. Of the pathogenic variants, 293 are indels (ins, dup, del, delins) and 497 were substitutions including 51 splice site variants. The small number of curated variant relative to the overall number of variants reflect the large numbers of technical artefacts found in NGS as well as the small number of genes in the cancer panel assays (Table 1).

Bioinformatics pipelines contain many complex algorithms with a large range of parameters controlling their behaviours. These parameters are typically set at the time of pipeline execution. A key design aim of PathOS is to provide web tools for downstream filtering giving the curation user interactive control of variant filtering and behaviour. For example, in our clinical pipelines, all called variants are passed unfiltered into PathOS where complex filtering can be applied on any variant attribute rather than the pipeline filtering in an opaque fashion.

## Conclusions

Clinical molecular diagnostics for cancer is presently undergoing a transformation driven by the widespread availability of an affordable generation of high throughput sequencers, which can describe a patient’s genetic data in nucleotide level detail. This holds the promise of a step change in our understanding of the impact of cancer biology on patient care. The realisation of this promise in the diagnostic lab has been limited by the lack of quality decision support tools that can interpret the output of sequencers and produce integrated reports suitable for a clinical audience.

PathOS is the response of our laboratory to this need and addresses many of the issues cited in our previous paper [1]. Built with commercial software engineering standards, it has been shown to be robust through two years of production diagnostic use in a rapidly evolving environment.

A key goal of the system is to minimise the time-consuming expert curation effort required for each novel variant. Curation throughput can be significantly improved by minimising the number of variants in the grey area between clearly reportable and clearly benign. By providing the decision support tools and evidence needed by curators to classify variants, the clinical turnaround time of diagnostic reporting can be reduced to the benefit of patients and clinicians.

PathOS is actively being developed and future effort will involve: improving the curation bottleneck to improve diagnostic throughput; scaling the system to accommodate a wider range of capture technologies; larger gene panels; and better visualisation of complex variants such as copy number variants and structural variants.

## Availability and requirements

Project Name: PathOS

Project Home Page: https://www.petermac.org/about/signature-centres/centre-clinical-cancergenomics/molecular-diagnostic-software

Project Repository: https://github.com/PapenfussLab/PathOS

Operating System(s): Docker compatible OS eg (Linux,Mac,AWS,Azure,Windows)

Programming Languages: Groovy, Java

Other requirements: Reference data

License: GNU license - GPL 3.0

## Additional files

Additional file 1: Figure S1. PathOSAnalysisWorkflow.pdf: Schematic of the typical data analysis workflow from sequencer to diagnostic pathology report. (PDF 65 kb)

Additional file 2: Table S1. AnnotationMetaData.xlsx: List of variant attributes kept in PathOS annotation cache. List of data sources used to annotate variants. (XLSX 17 kb)

Additional file 3: NGS Report Template.docx: Example MSWord Mail Merge template for PathOS reporting. (DOCX 674 kb)

## Abbreviations

API: Application programming interface
CI: Continuous integration
CNV: Copy number variants
CLIA: Clinical Laboratory Improvement Amendments
FFPE: Formalin-fixed paraffin-embedded
HGVS: Human Genome Variant Society
indel: Insertion / deletion
LDAP: Lightweight directory access protocol
LIMS: Laboratory Information Management System
LSDB: Locus-specific database
NATA: National Association of Testing Authorities
NGS: Next-generation sequencing
QC: Quality control
RBAC: Role-based access control
SNV: Single nucleotide variant
SOAP: Service-oriented architecture protocol
SOP: Standard operating procedures
SV: Structural variants
TCGA: The Cancer Genome Atlas
UAT: User acceptance testing
UI: User interface
WGS: Whole-genome sequencing
