# Supplementary figures and images for: PathOS: a decision support system for reporting high throughput sequencing of cancers in clinical diagnostic laboratories
Source: Genome Med. 2017 Apr 24;9:38. doi: 10.1186/s13073-017-0427-z (PMC5404673; doi:10.1186/s13073-017-0427-z)

# PathOS Analysis Workflow

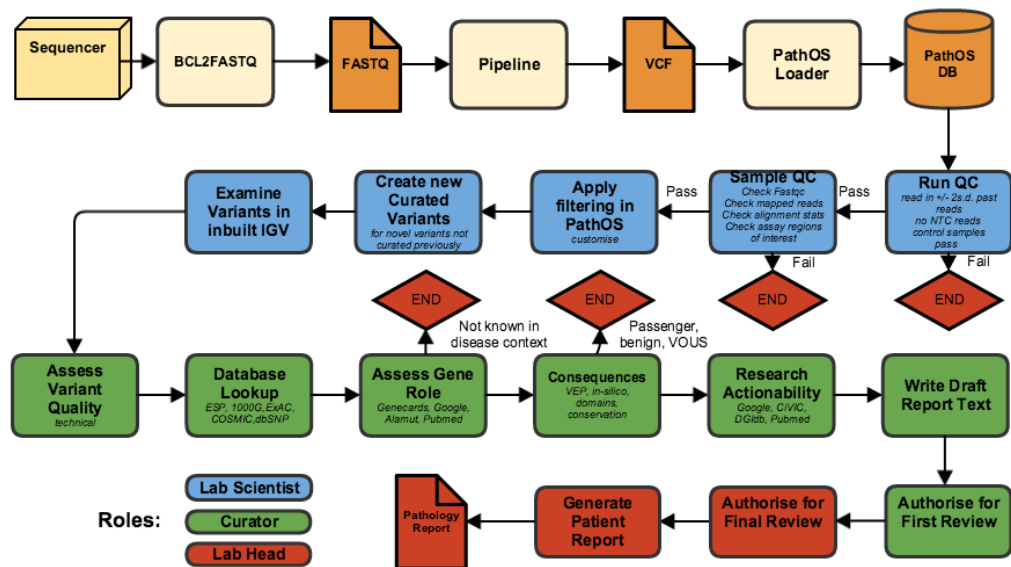

Supplement: Supplementary file 1 — PathOSAnalysisWorkflow.pdf: Schematic of the typical data analysis workflow from sequencer to diagnostic pathology report. (PDF 65 kb) [file 13073_2017_427_MOESM1_ESM.pdf]
