# Supplementary material for: PathOS: a decision support system for reporting high throughput sequencing of cancers in clinical diagnostic laboratories
Source: Genome Med. 2017 Apr 24;9:38. doi: 10.1186/s13073-017-0427-z (PMC5404673; doi:10.1186/s13073-017-0427-z)
Supplement: Supplementary file 3 — NGS Report Template.docx: Example MSWord Mail Merge template for PathOS reporting. (DOCX 674 kb) [file 13073_2017_427_MOESM3_ESM.docx]

«TableStart:Samples»

| To: PETER MAC CANCER CENTRE  ST ANDREWS PL  EAST MELBOURNE  VIC 3002 | **Patient**: «patient»  **URN**: «urn»  **DOB**: «dob»  **SEX**: «sex»  **Location**: «location»  **Requester**: «requester» | Sample: «sample»  Ext Ref: «extref»  Collected: «collect_date»  Received: «rcvd_date»  Specimen:  Block ID: |
| --- | --- | --- |
|  |  |  |

**NEXTGEN SEQUENCING AMPLICON PANEL REPORT**

**Specimen Details**

Block ID: «blockid», Tumour site: «site» Histology/Morphology: «morphology», Tumour cell content: «tumour_pct» % tumour estimated in marked area.

**Results**

| **Gene** | **Reference** | **Nucleotide Change** | **Inferred Protein Change** | **Read Depth^¶^** | **Classification** |
| --- | --- | --- | --- | --- | --- |
| **«TableStart:Variants»** **«gene»** | «refseq» | «hgvsc» | «hgvsp» | «varreaddepth»/«totalreaddepth » «afpct»% | «class» «TableEnd:Variants» |

^¶^ variant reads / total reads

**Interpretation**

«TableStart:Variants»**«gene»:** «mut»«TableEnd:Variants»

**Methods**

Tumour DNA is analysed using the Illumina TruSeq Custom Amplicon Cancer Panel, which targets the mutation hotspots of 48 cancer genes in 212 targeted amplicons. Samples are uniquely indexed, pooled and sequenced on the Illumina MiSeq using MiSeq v2 chemistry at 2x151bp reads. Alignment, variant calling and annotation are performed using Peter Mac’s amplicon-optimised pipeline v1.0. Only plausible pathogenic variants passing multiple functional and quality filters are reported above. Amplicons with less than 100 aligned reads are not analysed. These are listed below.

This mutation panel is designed to detect single nucleotide variants and indels in the target regions only. Mutations in the 48 cancer genes that lie outside the target regions will not be detected. The technology employed here is not suitable for detecting loss of heterozygosity, copy number variations, gross structural rearrangements, or aneuploidies. At 1000x coverage, this assay has a detection limit of approximately 5%. The variants detected by this assay should be confirmed by a second method before being used to guide clinical decisions.

**Comments**

DNA extraction of this tissue sample produced sufficient good quality material for TruSeq Amplicon testing. Sample processing passed all expected QC metrics and high quality sequence with high coverage («ampReads» mean aligned reads/amplicon) and uniformity («ampPct» % amplicons >0.2 mean aligned reads) was obtained.

«TableStart:Variants»«genedesc»«TableEnd:Variants»

Please contact the laboratory on 03 9734 4565 if you wish to discuss this report further.

**This test has not yet been fully validated to the current NPAAC requirements for an in-house IVD and results should be interpreted accordingly. All findings should be confirmed by an independent clinical assay. For further information, please contact the laboratory.**

Reported by: Dr A Asimov, Scientist in Charge Molecular Pathology Diagnostic Development

Authorised by: Prof S Pathohead, Director of Pathology

Reported: 11-Jul-2016 12:37 pm

Low quality amplicons:

«lowAmps»

Regions of interest coverage:

«rois»

References:

«TableStart:Variants»«refs»«TableEnd:Variants»

*«TableEnd:Samples»*
